# Meta-analysis of whole-genome gene expression datasets assessing the effects of *IDH1* and *IDH2* mutations in isogenic disease models

**DOI:** 10.1038/s41598-021-04214-7

**Published:** 2022-01-07

**Authors:** Hans-Juergen Schulten, Fatima Al-Adwani, Haneen A. Bin Saddeq, Heba Alkhatabi, Nofe Alganmi, Sajjad Karim, Deema Hussein, Khalid B. Al-Ghamdi, Awatif Jamal, Jaudah Al-Maghrabi, Mohammed H. Al-Qahtani

**Affiliations:** 1 Center of Excellence in Genomic Medicine Research, Department of Medical Laboratory Technology, Faculty of Applied Medical Science, King Abdulaziz University, P.O. Box 80216, Jeddah, 21589 Saudi Arabia; 2 Department of Medical Laboratory Technology, Faculty of Applied Medical Science, King Abdulaziz University, Jeddah, Saudi Arabia; 3 Department of Computer Science, King Abdulaziz University, Jeddah, Saudi Arabia; 4 King Fahad Medical Research Center, Department of Medical Laboratory Technology, Faculty of Applied Medical Science, King Abdulaziz University, Jeddah, Saudi Arabia; 5 Department of Otolaryngology, Head and Neck Surgery, Faculty of Medicine, King Abdulaziz University, Jeddah, Saudi Arabia; 6 Department of Pathology, Faculty of Medicine, King Abdulaziz University, Jeddah, Saudi Arabia; 7 Department of Pathology, King Faisal Specialist Hospital and Research Center, Jeddah, Saudi Arabia

**Keywords:** Cancer genetics, Data mining, Cancer, Genetics, Molecular biology

## Abstract

Mutations in isocitrate dehydrogenase 1 (*IDH1*) and *IDH2* are oncogenic drivers to a variable extent in several tumors, including gliomas, acute myeloid leukemia (AML), cholangiocarcinoma, melanoma, and thyroid carcinoma. The pathobiological effects of these mutations vary considerably, impeding the identification of common expression profiles. We performed an expression meta-analysis between IDH-mutant (IDH^mut^) and IDH-wild-type (IDH^wt^) conditions in six human and mouse isogenic disease models. The datasets included colon cancer cells, glioma cells, heart tissue, hepatoblasts, and neural stem cells. Among differentially expressed genes (DEGs), serine protease 23 (*PRSS23*) was upregulated in four datasets, i.e., in human colon carcinoma cells, mouse heart tissue, mouse neural stem cells, and human glioma cells. Carbonic anhydrase 2 (*CA2*) and prolyl 3-hydroxylase 2 (*P3H2*) were upregulated in three datasets, and SOX2 overlapping transcript (*SOX2-OT*) was downregulated in three datasets. The most significantly overrepresented protein class was termed intercellular signal molecules. An additional DEG set contained genes that were both up- and downregulated in different datasets and included oxidases and extracellular matrix structural proteins as the most significantly overrepresented protein classes. In conclusion, this meta-analysis provides a comprehensive overview of the expression effects of IDH mutations shared between different isogenic disease models. The generated dataset includes biomarkers, e.g., PRSS23 that may gain relevance for further research or clinical applications in IDH^mut^ tumors.

## Introduction

Isocitrate dehydrogenases (IDHs) consist of three isozymes, i.e., IDH1, IDH2, and IDH3, which are key metabolic enzymes catalyzing the conversion of isocitrate to α-ketoglutarate (α-KG) via oxidative decarboxylation. IDH1 is located in the cytosol and peroxisomes, whereas IDH2 and IDH3 are located in the mitochondria. As components of the citrate acid cycle (CAC), IDH1 and IDH2 use NADP + as a coenzyme, whereas IDH3 uses NAD + as a coenzyme. The generated NADPH and NADH are reducing equivalents necessary for diverse metabolic and physiological processes.

Recurrent *IDH1* mutations affecting codon R132 were initially identified in glioblastoma multiforme (GBM), where the mutation showed a significant prevalence in secondary GBM^1^. Subsequently, *IDH2* mutations affecting codon R172, which is homologous to *IDH1* R132, were detected in WHO grade II and III astrocytomas and oligodendrogliomas as well as in secondary GBM^2^. Virtually all *IDH1* mutations in gliomas affect codon R132, which in the vast majority (> 85%) is a heterozygous missense mutation of arginine to histidine (R132H)^3^. Other less frequent *IDH1* R132 mutations leading to different amino acid replacements, including R132C, R132G, R132G, and R132L, have been described in a number of solid and hematopoietic neoplasms and related pathogenic processes^4,5^. In anaplastic thyroid carcinoma, *IDH1* mutations are relatively common and affect the highly conserved residue G123^6,7^. In acute myeloid leukemia (AML), *IDH2* mutations are more prevalent than *IDH1* mutations and usually affect codon R140. No oncogenic *IDH3* mutations have been reported so far.

The oncogenic capacity of IDH1/2 mutations is conferred by a catalytically active dimer, most likely consisting of an IDH-mutant (IDH^mut^) and an IDH-wild-type (IDH^wt^) heterodimer, which reduces α-KG to D-2-hydroxyglutarate (D-2HG)^8,9^. D-2HG is an oncometabolite that induces diverse metabolic and cellular effects, e.g., affecting CAC, inhibiting α-KG-dependent enzymes, such as histone and DNA demethylases, and blocking transcriptionally regulated cellular differentiation^4,10–12^. In particular, a DNA methylation profile is induced that varies between different IDH^mut^ tumor types. For example, gliomas exhibit a DNA methylation profile, referred to as a glioma cytosine-phosphate-guanine (CpG) island methylator phenotype (G-CIMP), which differs from those in AML, cholangiocarcinoma, and melanoma^13–15^. The discrepancies observed in the DNA methylation profiles of the investigated tumor types are also observed in the transcriptional profiles impeding the ability to assess common effects of IDH mutations on the transcriptome. Research on epigenetic and transcriptional effects of IDH mutations in cancer is ongoing, e.g., a recent study reported that transcriptional alterations in IDH1^mut^ glioma samples are primarily caused by chromatin-based DNA methylation-independent mechanisms^16^.

IDH mutations represent a valuable target for cancer treatment because they are commonly associated with early oncogenesis and are retained through later cancer stages. However, the success of therapy strategies varies between different IDH^mut^ tumor types, and alternative treatment options, such as the application of glutaminase inhibitors, are assessed^17–19^. Isogenic disease models have become a valuable method in cancer research and drug discovery for studying the effects of a particular gene mutation in comparison to otherwise genetically identical cells^20^. In particular, isogenic disease models have been repeatedly used to determine the transcriptional effects of IDH mutations under nearly unbiased conditions. We therefore performed a meta-analysis on datasets that compared expression profiles between IDH^mut^ and IDH^wt^ isogenic disease models with the aim of identifying biomarkers that have prospects for research or clinical applications.

## Results

### Compilation of datasets

The meta-analysis included six studies that were extracted from a database search and that compared the expression profiles between IDH1/2^mut^ and IDH1/2^wt^ conditions in isogenic disease models (Table 1). Four studies utilized microarrays, one used BeadChips, and one used RNA-sequencing (RNA-seq) to generate sets of differentially expressed genes (DEGs). In two studies, expression experiments were performed using human cell lines. In four studies, microarray expression experiments were performed using mouse cells/cell lines, mouse tissues or tumors. DEGs were determined based on a false discovery rate (FDR)-adjusted *p*-value ≤ 0.05 and a fold change (FC) ≥ 1.5.

**Table 1:** Expression studies on isogenic disease models included in the meta-analysis.

GEO dataset	Cells/tissue origin	No. of samples		Platform	No. of DEGs^a^	Year/ref.
		IDH^mut^	IDH^wt^			
GSE96979	Mouse glioma cells	3 IDH1^R132H^	2 IDH1^wt^	Illumina MouseWG-6 v2.0 expression BeadChip	250-500	2017^21^
GSE41802	Human HCT116 colon carcinoma cells^b^	4 IDH1^R132H^ 2 IDH1^R132C^ 4 IDH2^R172K^	2 IDH1/2^wt^	Affymetrix Human Genome U133 Plus 2.0 Array	> 500	2012^22^
GSE54838	Mouse heart tissue	4 IDH2^R140Q^ 4 IDH2^R172K^	4 IDH2^wt^	Affymetrix Mouse Gene 1.0 ST Array	250-500	2014^23^
GSE57002	Mouse hepatoblasts^c^	2 IDH1^R132C^ 2 IDH2^R172K^	2 IDH1^wt^ 2 IDH2^wt^	Affymetrix Mouse Genome 430A 2.0 Array	50-250	2014^24^
GSE88828	Mouse neural stem cells	3 IDH1^R132H^	3 IDH1^wt^	Affymetrix Mouse Gene 2.0 ST Array	50-250	2017^25^
GSE147223	Human U251 glioma cells	3 IDH1^R132H^ 3 IDH1^R132C^	3 IDH1^wt^	Illumina HiSeq 2500	250-500	2020^26^

^a^For the four mouse isogenic disease models, the percentage of mouse genes without corresponding human ortholog ranged between approximately 8.6% (mouse hepatoblasts), 12.8% (mouse neural stem cells), 17.1% (mouse heart tissue), and 18.7% (mouse glioma cells). ^b^Low 2-HG expressing IDH2^R140^^Q^ cells were excluded from analysis and the respective parental cells were used as control. ^c^The batch grown on uncoated plates for hepatocyte differentiation was excluded from analysis.

### Genes either up- or downregulated in the meta-analysis dataset

**Figure 1:**
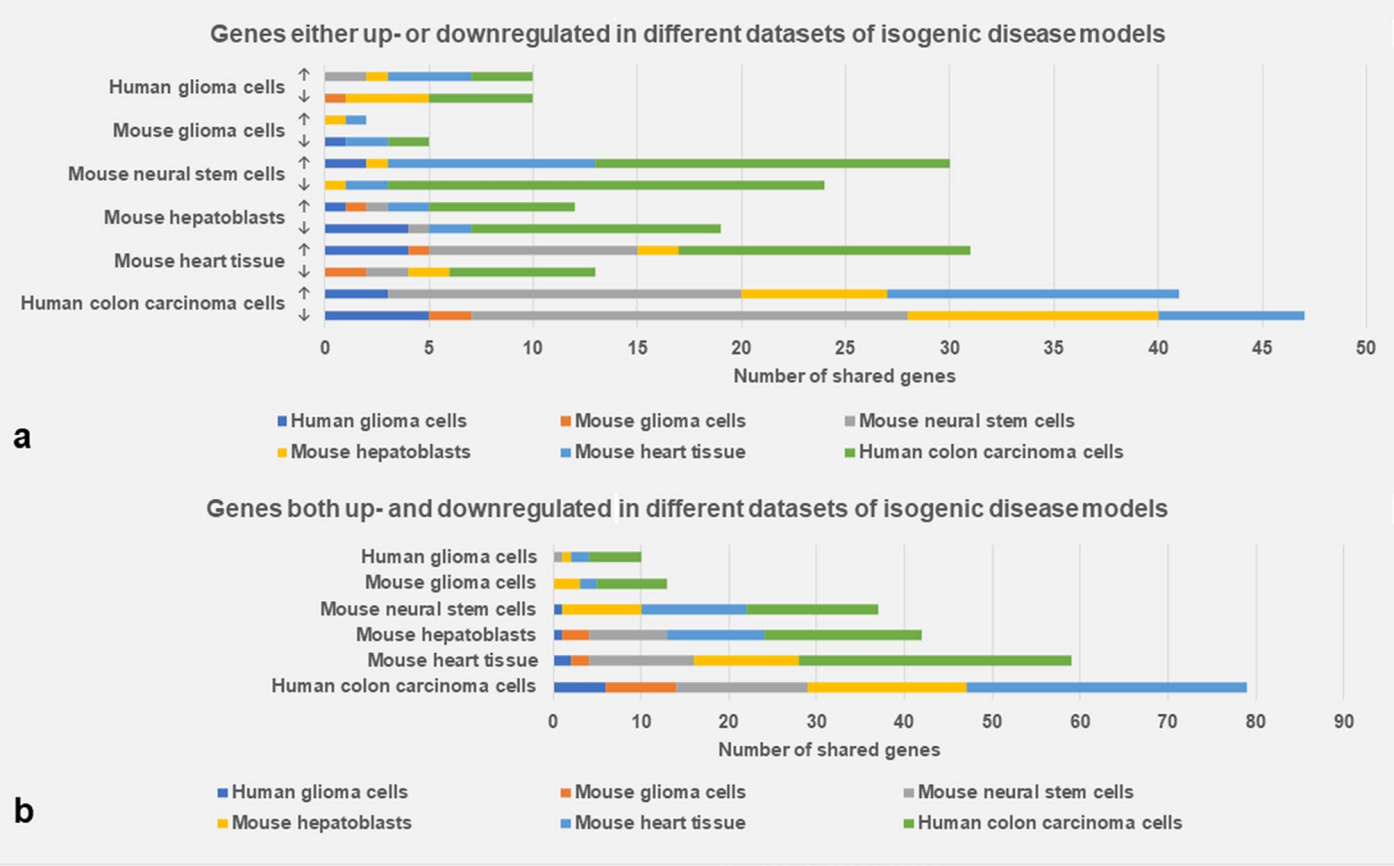
Bar charts illustrating the number of genes shared between at least two of the six analyzed DEG sets of the isogenic disease models. (**a**) Genes, which are either up- (↑) or downregulated (↓) in different datasets. (**b**) Genes, which are both up- and downregulated in different datasets.

The number of DEGs in the individual datasets and the proportion of DEGs that were either up- or downregulated between at least two datasets varied considerably between the studies (Table 1, Fig. 1a). For example, in relation to the number of DEGs in individual datasets, mouse glioma cells shared proportionally fewer genes while mouse neural stem cells shared proportionally more genes with the common DEG set. The shared dataset comprised 111 DEGs, of which 49% were upregulated and 51% were downregulated (Table 2). Serine protease 23 (*PRSS23*) was upregulated in four datasets, i.e., human colon carcinoma cells, mouse heart tissue, mouse neural stem cells, and human glioma cells. Carbonic anhydrase 2 (*CA2*), and prolyl 3-hydroxylase 2 (*P3H2*) were upregulated in three datasets, whereas SOX2 overlapping transcript (*SOX2-OT*) was downregulated in three datasets.

**Table 2:** Meta-analysis DEG sets compiled from individual datasets of isogenic disease models.

Deregulated genes shared between datasets of isogenic disease models
**Genes either up- or downregulated**
Upregulated genes
ARPC5, BDH1, BDNF, BMP4, CA2, CACHD1, CACNB4, CCDC80, CHST11, CHST15, CLU, CSF1, CYBRD1, DHX37, DPYSL5, EPAS1, EPDR1, FAM189A1, FOXF1, FSCN1, GALNS, GIMAP6, GRK5, HMOX1, KBTBD8, KIF3C, LGR4, MAML2, MANEAL, MAP1A, MCAM, MEST, MYBL1, OTUB2, P3H2, PLD1, PODXL, PRSS23, RCAN1, S100A2, SCARB1, SDC1, SEMA7A, SEPTIN11, SERINC2, SLC38A3, SPRY1, STC1, STK17A, TBC1D4, TGFB2, WNT7A, ZBTB7C, ZGRF1
Downregulated genes
ABCA12, ARRDC4, AZGP1, CACNA2D1, CAPN6, Ccl9, CFB, COL3A1, COL6A3, CRYL1, CTNNA3, Cyp3a13 (related to human CYP3A7),DHDH, DHRS7, ENPP2, F3, FBLIM1, GPC3, GPT2, HERPUD1, IFITM1, IFITM2, INAVA, KIZ, KLHDC1, KRT20, LARP1B, LNCAROD, LONRF2, LRATD2, LRG1, MACROD1, MIA2, MMRN2, NT5DC2, PCDH10, PCDH7, PCOLCE, PDK1, PLEKHH1, PPL, PPM1K, PRPH, PYCR1, RIMKLB, RPS6KA5, RTN2, SERPINH1, SLC7A11, SNCA, SOCS2, SOX2-OT, SPINT1, STRA6, TCAIM, TFPI, TXNIP
**Genes both up- and downregulated**
ADGRG1, AGPAT5, AMOT, ANK1, ANKRD1, ANTXR2, ANXA2, APOL6, ARMH4, BCL2L11, BHLHE40, CCN2, CDC42EP3, CDO1, CDS1, CELSR2, COL4A1, COL4A2, COL8A1, CTSH, CXCR4, CYP1B1, DNAH2, DRD2, DUSP5, ELFN2, EMP1, EPB41L4B, ERAP1, FBLN1, FBN1, FN1, FRAS1, GCNT1, GPRC5B, HAS2, HDHD2, HIVEP2, HSPA5, IDH2, IGFBP4, IGFBP7, ISG20, KDELR3, KDM5B, KLHL32, LIPH, LMCD1, LMNA, LOX, MACROD2, MCM5, MFSD2A, MGP, MTCL1, MYBL2, MYT1, NEBL, NOSTRIN, NR4A1, NR4A3, NRG2, P4HA2, PDLIM3, PER3, PITPNC1, PKMYT1, PLAUR, PLPP2, PRODH, PRSS35, QSOX1, QSOX2, RGMA, SERPINE1, SH3GL3, SLC16A2, SLC1A4, SLC25A28, SLC26A6, SLC2A1, SLC2A12, SNAP25, SOX2, SPP1, SYNPO, TEF, TGFBI, TGFBR2, TGFBR3, TGM2, TKTL1, TNC, TPM2, TRNP1, UPP1, VEGFA, WDR90

### Ontology and pathway analysis of genes either up- or downregulated in the meta-analysis dataset

**Figure 2:**
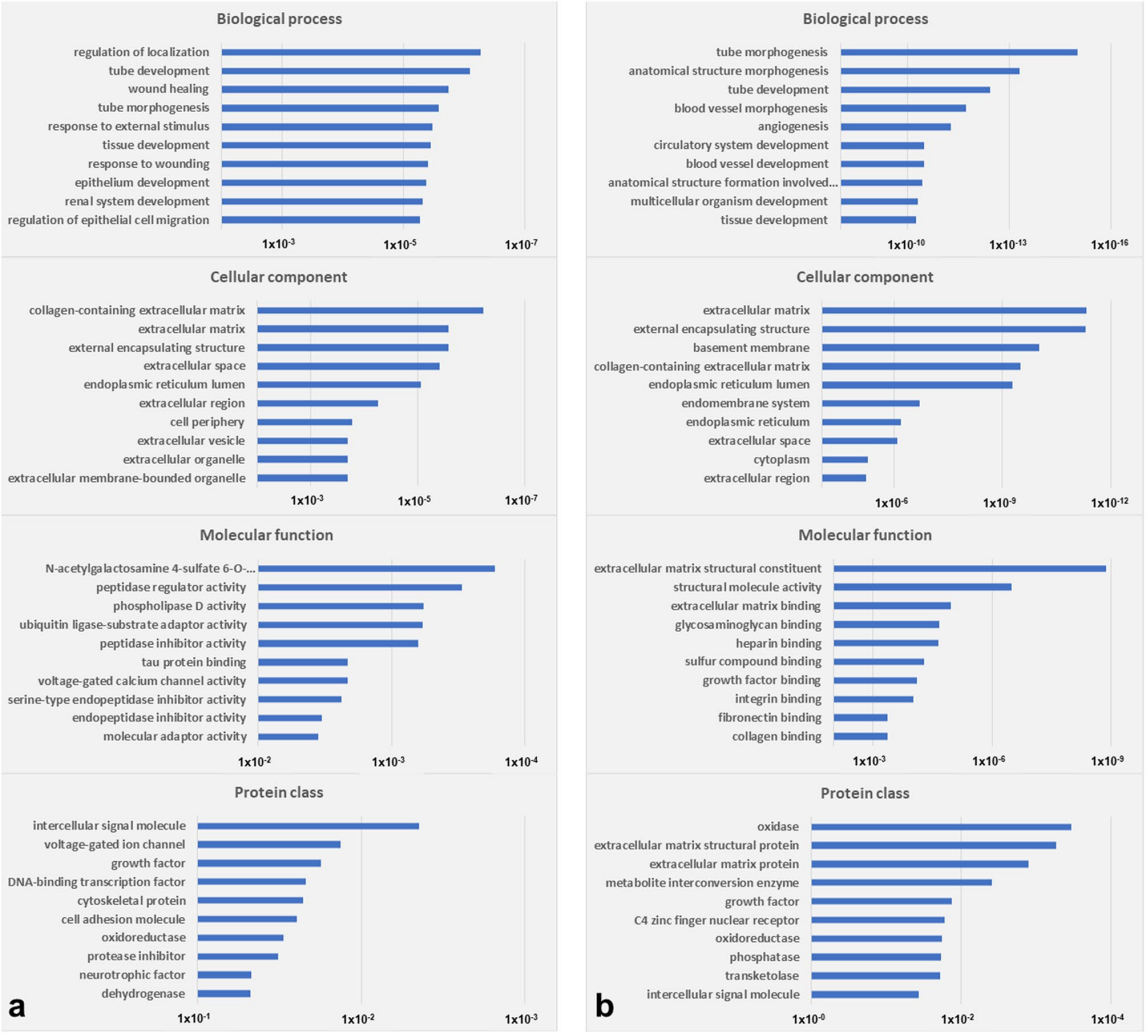
GO annotations in the categories of biological process, cellular component, and molecular function, and protein class ontology annotations. (**a**) Genes either up- or downregulated in the meta-analysis dataset compiled from isogenic disease models. (**b**) Genes both up- and downregulated in the meta-analysis dataset compiled from the isogenic disease models. A Fisher’s exact test *p*-value < 0.05 indicated statistical significance.

**Figure 3:**
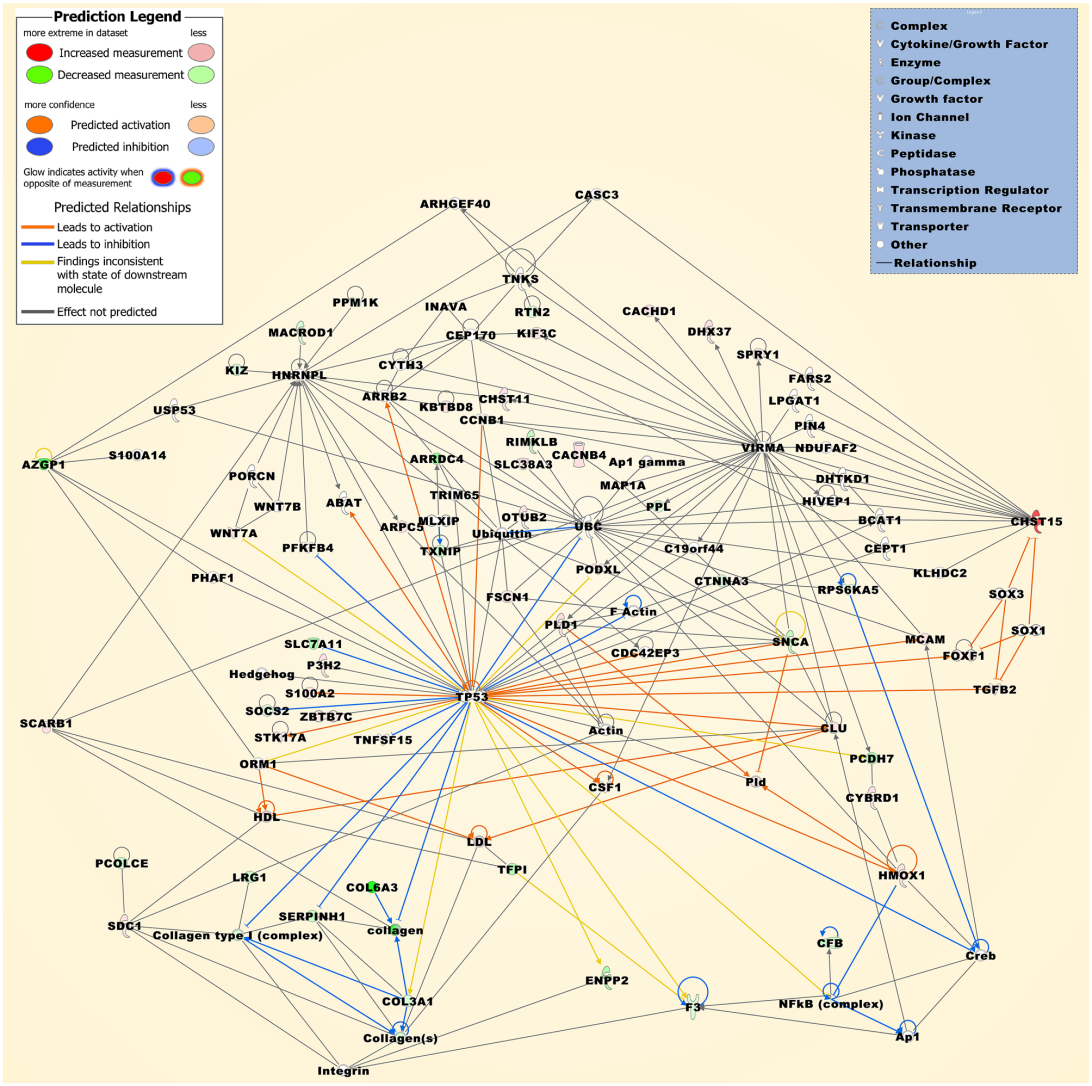
The merged network is compiled from the top three networks that were most significantly associated with the DEGs, which were either up- or downregulated in at least two individual datasets (Table 3). Upregulated molecules include ARPC5, CACHD1, CACNB4, CHST11, CHST15, CLU, CSF1, CYBRD1, DHX37, FOXF1, FSCN1, HMOX1, KBTBD8, KIF3C, MAP1A, MCAM, OTUB2, PLD1, PODXL, S100A2, SCARB1, SDC1, SLC38A3, SPRY1, STK17A, TGFB2, WNT7A, and ZBTB7C. Downregulated molecules include ARRDC4, AZGP1, CFB, COL3A1, COL6A3, CTNNA3, ENPP2, F3, INAVA, KIZ, LRG1, MACROD1, P3H2, PCDH7, PCOLCE, PPL, PPM1K, RIMKLB, RPS6KA5, RTN2, SERPINH1, SLC7A11, SNCA, SOCS2, TFPI, and TXNIP. Molecular relationship factors were added from the Ingenuity knowledge base comprising ABAT, Actin, Ap1, Ap1 gamma, ARHGEF40, ARRB2, BCAT1, C19orf44, CASC3, CCNB1, CDC42EP3, CEP170, CEPT1, collagen, Collagen type I (complex), Collagen(s), Creb, CYTH3, DHTKD1, F Actin, FARS2, HDL, Hedgehog, HIVEP1, HNRNPL, Integrin, KLHDC2, LDL, LPGAT1, MLXIP, NDUFAF2, NFkB (complex), ORM1, PFKFB4, PHAF1, PIN4, Pld, PORCN, S100A14, SOX1, SOX3, TNFSF15, TNKS, TP53, TRIM65, UBC, Ubiquitin, USP53, VIRMA, and WNT7B. The molecule activity predictor was implemented to display further molecular effects as itemized in the prediction legend.

The most significantly overrepresented gene ontology (GO) annotations in the DEG set included diverse morphogenic and developmental processes, extracellular matrix and organelle components, and molecular activities in the categories of biological process, cellular component, and molecular function, respectively (Fig. 2a). The most significantly  overrepresented protein class (*p* = 2.04 × 10^–3^) was related to intercellular signal molecules (Fig. 2a) and included BDNF, BMP4, RCAN1, SEMA7A, STC1, TGFB2, and WNT7A, all of which were comparably upregulated under IDH1/2^mut^ conditions. The most significantly overrepresented pathways included extracellular matrix organization, collagen biosynthesis and modifying enzymes, and collagen formation (Table 3). The most significantly associated networks were related to various diseases, conditions, and cellular functions (Table 3). The top three networks were assembled with molecular relationship factors and displayed as a merged network (Fig. 3). Further interpretation of the DEG set was performed with the upstream regulator analysis tool (Supplementary Fig. 1). Activated upstream regulators that were predicted to be most significantly associated with the DEG set comprised chorionic gonadotropin (CG) complex, cytokine WNT3A, transmembrane receptor IL10RA, and transcription factor TP53. The transporter APOE and cytokine IFNG were predicted to be the most significantly inhibited upstream regulators.

**Table 3:** Top pathways and networks compiled from the meta-analysis DEG sets.

Category	*p*-values	Score
**Top Reactome pathways**		
Genes either up- or downregulated		
Extracellular matrix organization	3.96x10^-5^	
Collagen biosynthesis and modifying enzymes	4.07x10^-5^	
Collagen formation	1.46x10^-4^	
Chondroitin sulfate/dermatan sulfate	1.81x10^-4^	
NCAM signaling for neurite out-growth	3.53x10^-4^	
Genes both up- and downregulated		
Integrin cell surface interactions	2.7010x^-7^	
Extracellular matrix organization	2.79x10^-7^	
Post-translational protein phosphorylation	1.25x10^-6^	
Regulation of insulin-like growth factor (IGF) transport and uptake by insulin-like growth factor binding proteins (IGFBPs)	3.18x10^-6^	
ECM proteoglycans	4.35x10^-5^	
**Top IPA networks**		
Genes either up- and downregulated		
Cancer, cellular movement, organismal injury and abnormalities		48
Cancer, organismal injury and abnormalities, tissue morphology		33
Amino acid metabolism, molecular transport, small molecule biochemistry		28
Developmental disorder, hereditary disorder, ophthalmic disease		25
Nervous system development and function, tissue morphology, cell morphology		25
Genes both up- and downregulated		
Cellular development, cellular growth and proliferation, cancer		50
Cardiovascular system development and function, organismal development, tissue development		44
Neurological disease, nucleic acid metabolism, small molecule biochemistry		26
Amino acid metabolism, small molecule biochemistry, cancer		19
Cell-to-cell signaling and interaction, cardiovascular system development and function, hereditary disorder		19

### Genes both up- and downregulated in the meta-analysis dataset

An additionally shared DEG set comprised 98 genes that were both up- and downregulated in two or three individual datasets (Fig. 1b; Table 2). Genes both up- and downregulated in three datasets included armadillo like helical domain containing 4 (*ARMH4*), cellular communication network factor 2 (*CCN2*), erythrocyte membrane protein band 4.1 like 4B (*EPB41L4B*), fibulin 1 (*FBLN1*), fibronectin 1 (*FN1*), G protein-coupled receptor class C group 5 member B (*GPRC5B*), serine protease 35 (*PRSS35*), serpin family E member 1 (*SERPINE1*), solute carrier family 16 member 2 (*SLC16A2*), secreted phosphoprotein 1 (*SPP1*), and synaptopodin (*SYNPO*).

### Ontology and pathway analysis of genes both up- and downregulated in the meta-analysis dataset

**Figure 4:**
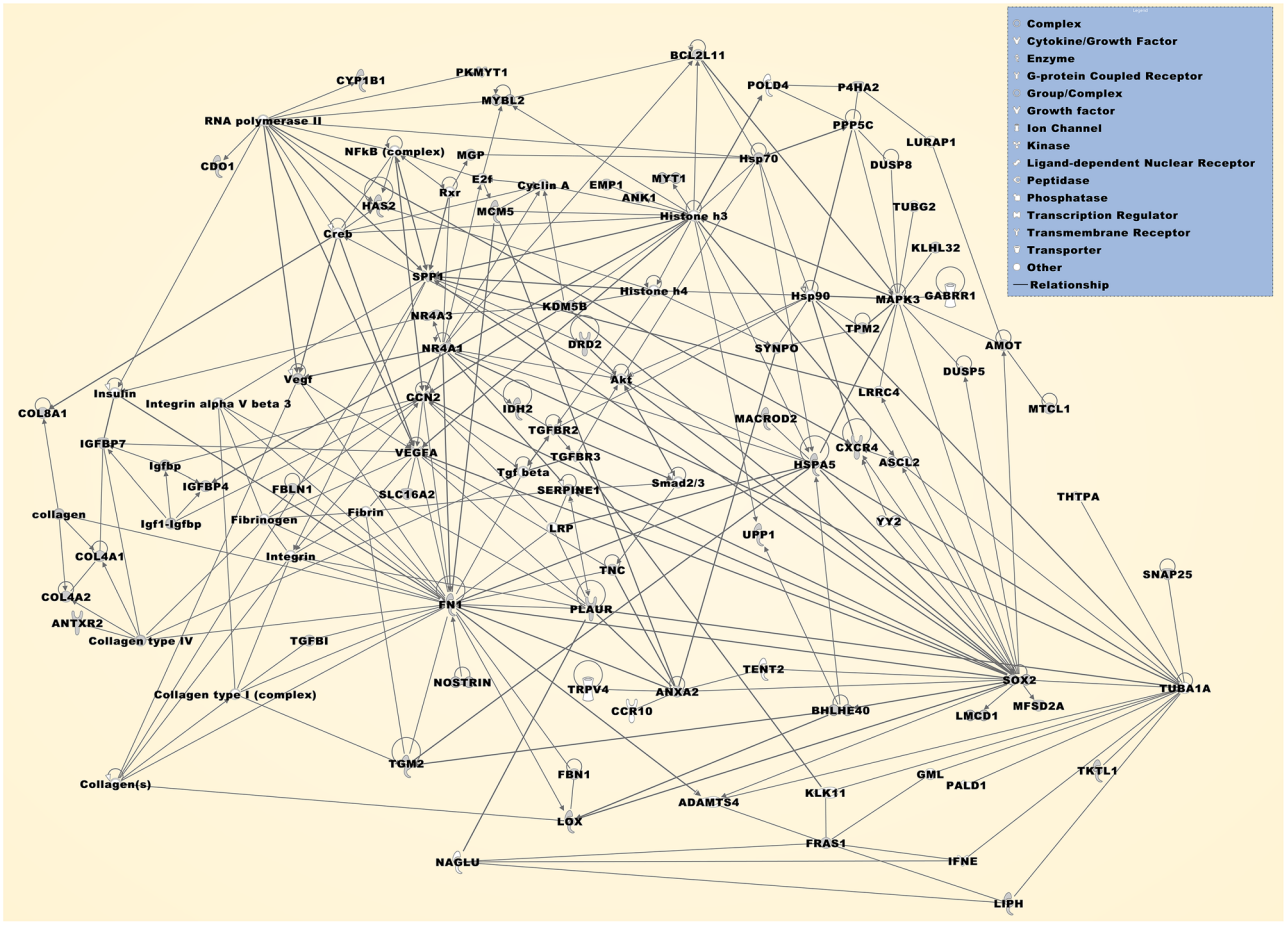
The merged network is compiled from the top three networks that were most significantly associated with the DEGs, which were both up- and downregulated in at least two individual datasets (Table 3). Deregulated molecules comprise AMOT, ANK1, ANTXR2, ANXA2, BCL2L11, BHLHE40, CCN2, CDO1, COL4A1, COL4A2, COL8A1, CXCR4, CYP1B1, DRD2, DUSP5, EMP1, FBLN1, FBN1, FN1, FRAS1, HAS2, HSPA5, IDH2, IGFBP4, IGFBP7, KDM5B, KLHL32, LIPH, LMCD1, LOX, MACROD2, MCM5, MFSD2A, MGP, MTCL1, MYBL2, MYT1, NOSTRIN, NR4A1, NR4A3, P4HA2, PKMYT1, PLAUR, SERPINE1, SLC16A2, SNAP25, SOX2, SPP1, SYNPO, TGFBI, TGFBR2, TGFBR3, TGM2, TKTL1, TNC, TPM2, UPP1, and VEGFA. Molecular relationship factors were added from the Ingenuity knowledge base comprising ADAMTS4, Akt, ASCL2, CCR10, collagen, Collagen type I (complex), Collagen type IV, Collagen(s), Creb, Cyclin A, DUSP8, E2f, Fibrin, Fibrinogen, GABRR1, GML, Histone h3, Histone h4, Hsp70, Hsp90, IFNE, Igf1-Igfbp, Igfbp, Insulin, Integrin, Integrin alpha V beta 3, KLK11, LRP, LRRC4, LURAP1, MAPK3, NAGLU, NFkB (complex), PALD1, POLD4, PPP5C, RNA polymerase II, Rxr, Smad2/3, TENT2, Tgf beta, THTPA, TRPV4, TUBA1A, TUBG2, Vegf, and YY2.

The most significantly overrepresented GO annotations in the DEG set included diverse morphogenic and developmental processes, extracellular matrix components, and various binding properties in the categories of biological process, cellular component, and molecular function, respectively (Fig. 2b). The most significantly overrepresented protein classes included oxidases (*p* = 3.36 × 10^–4^), comprising PRODH, LOX, QSOX1, and QSOX2, and extracellular matrix structural proteins (*p* = 5.36 × 10^–4^) comprising COL4A1, COL4A2, COL8A1, and FBN1 (Fig. 2b). The most significantly overrepresented pathways included integrin cell surface interactions, extracellular matrix organization, and post-translational protein phosphorylation (Table. 3). The most significantly associated networks were related to various developmental processes, diseases, conditions, and cellular functions (Table 3). The top three networks were assembled with molecular relationship factors and displayed as a merged network (Fig. 4).

## Discussion

In this meta-analysis, we compared the expression profiles of different IDH^mut^ *vs*. IDH^wt^ isogenic disease models to provide an overview of the nearly unbiased expression effects and the corresponding biological interpretations caused by the oncometabolite 2-HG. Although the statistical power of the IDH^mut^ *vs*. IDH^wt^ isogenic cell model datasets is generally lower than that of larger datasets generated in clinical tumor cases, the number of DEGs in proportion to the sample size is seemingly higher in isogenic cell models^21^. One likely explanation for this fact is that individual expression profiles vary considerably within IDH^mut^ tumors, similar to as in other tumors, limiting the capacity to generate common expression profiles. However, in our meta-analysis, only a relatively low number of DEGs were shared between individual datasets, which can be attributed to the fact that different cancer and non-cancer isogenic disease models and experimental conditions were used as briefly outlined as follows: Using colon carcinoma cells, in which IDH1/2 mutations were inserted via a recombinant adeno-associated virus vector methodology, an epithelial-mesenchymal transition (EMT)-like phenotype and changes in gene expression and cell morphology were observed^22^. In transgenic mouse models with conditional IDH2^mut^ coding sequences, activation of IDH2^mut^ expression at five weeks of age produced D-2HG leading to cardiomyopathy and neurodegeneration^23^. In hepatoblasts, isolated from mouse embryos at E14, a doxycycline-inducible system led to IDH1/2^mut^ gene expression^24^. The IDH1/2^mut^ hepatoblasts, which were cultured on collagen-coated plates, were refractory to differentiation. In neural stem cells derived from the cortex of mouse embryos at E14.5, Idh1^mut^ expression was induced via adenoviral-Cre-recombinase transduction^25^. In these cells, neuronal lineage differentiation was blocked, although differentiation-promoting culture conditions were utilized. Employing a mouse model that is susceptible to the development of gliomas, p53-deficient cells with vector-integrated IDH1^mut^ genes and cells containing a PDGF expression vector were coinjected into mice. The induced PDGF-driven gliomas showed reduced immune infiltration in comparison to the corresponding IDH1^wt^ glioma mouse model^21^. In an in vitro study, glioma cells were infected with lentivirus IDH1^mut^ coding sequences^26^. Doxycycline-induced IDH1^mut^ gene expression resulted in enhanced cell motility and morphological changes. The heterogeneity between the six isogenic disease models is exemplarily demonstrated by the diverse classification of the top pathways that were derived from the DEGs of each of the disease models (Supplementary Fig. 2).

The serine protease PRSS23 exhibits low tissue specificity in humans with the highest expression levels in female genital tract tissue and smooth muscle^27^. Studies in mice reported that PRSS23 is variably expressed in the preimplantation uterus and is possibly involved in tissue remodeling in the ovary^28,29^. The expression of PRSS23 has been detected in nuclei and extracellular vesicular exosomes where the protease is a component of the human secretome^30^. Exosomal PRSS23 is, e.g., involved in cardiovascular disease where the protease likely mediates Snail/alpha‐smooth muscle actin signalling^31^. In cancer, PRSS23 is implicated in tumor progression, and it was identified in a systematic network survey of a meta-analysis of breast cancer microarray expression data as one of six genes involved in acquired lapatinib resistance^32^. Promoter studies in breast cancer cells indicated that *PRSS23* is upregulated by estrogen receptor 1 (ESR1) and that its upregulated expression contributes to cell proliferation^33^. shRNA-mediated knockdown of PRSS23 in a gastric cancer xenograft mouse model resulted in a decrease in tumor volume and tumor weight^34^. Further in vitro experiments revealed that PRSS23 knockdown in gastric cancer cells apparently affected EIF2 pathway molecules. Based on a microarray study, *PRSS23* was included in a gene classifier set that could discriminate papillary thyroid carcinoma from normal thyroid samples^35^. In head and neck, renal, and pancreatic cancer, PRSS23 expression is significantly associated with an unfavorable prognosis^30^. An epigenome-wide association study found, among several other DNA methylation sites, a significant association between changes of DNA methylation of DNA methylation sites at the *PRSS23* gene and having a smoking habit but found no significant association with risk for lung cancer^36^. The BioGRID database currently curates about 50 PRSS23 interactors, among which actin and actin-related proteins constitute the most overrepresented PANTHER protein class (*p*-value = 3 × 10^–3^) (Supplementary Fig. 3).

Cytosolic CA2 is the physiologically predominant CA isoform and is known to interact with various acid/base transporters^37^. These interactions are predicted to promote high glycolytic activity and cell proliferation in tumors. In lung cancer xenograft mouse models, shRNA-mediated knockdown of CA2 impaired tumor cell proliferation and angiogenesis and induced apoptosis^38^. Pharmacological studies exploring CA2 inhibitors are pursued to develop therapeutic options for the treatment of various conditions including cancer^39^. P3H family members consist of three isoenzymes in vertebrates. From a knockout study on *P3H2* in a mouse embryonal carcinoma cell line, it can be presumed that the enzyme is the major posttranslational modifier of type IV collagen with 3-hydroxyproline, which is of significance for interactions of type IV collagen with other molecules^40^. High P3H2 expression in different parts of the CNS, gastrointestinal tract, and some other tissues has been reported; however, the enzyme exhibits no prognostic significance in cancer and reveals only weak-to-moderate staining in most cancer tissues^30^. The long non-coding RNA (lncRNA) *SOX2-OT* consists of several splice variants. *SOX2*, located in an intron of SOX2-OT, is transcribed in the same orientation as SOX2-OT and both are intensely expressed in embryonic stem cells^41^. SOX2-OT is implicated in neuronal and tumor development and progression. A meta-analysis of cancer datasets indicated that cancers with elevated *SOX2-OT* expression are significantly associated with unfavorable prognostic factors^42^. In two cervical cancer cell lines, a *SOX2-OT* transcript variant promoted cell growth, migration and invasion of the cells, indicating that the lncRNA may constitute a practical biomarker for cervical cancer^43^. However, lower expression of *SOX2-OT* was observed in gastric tumors compared to matched normal gastric samples, and lower expression was observed in high-grade rather than low-grade gastric tumors^44^.

Furthermore, we assessed the similarity of expression profiles between the either up- or downregulated gene set from our meta-analysis with expression profiles of two publicly accessible datasets of low grade gliomas and chondrosarcomas, enabling us to compare IDH^mut^ with IDH^wt^ cancer samples^45–47^. The Venn diagram demonstrates that only a few DEGs are shared between our meta-analysis dataset and both clinical datasets (Supplementary Fig. 4). One likely explanation for this fact is that primary expression effects of an IDH mutation that emerge over days or weeks are measured in isogenic disease models, whereas clinical IDH^mut^ tumors evolve over months or years and acquire multiple other genomic alterations before they become clinically evident.

In summary, we generated a set of DEGs and biomarkers associated with IDH^mut^ status in isogenic disease models. Extracellular proteins and intercellular signaling are among the notable features of IDH^mut^ conditions. Biomarkers associated with various IDH^mut^ conditions, including the less characterized protease PRSS23, have considerable prospects for further research or clinical applications of IDH^mut^ cancers and related diseases.

## Methods

### Compilation of datasets from IDH1/2^mut^ vs. IDH1/2^wt^ isogenic disease models

Using the search term IDH to query the Gene Expression Omnibus (GEO), we designated 114 case series, out of which we identified seven whole-genome gene expression datasets derived from human and mouse isogenic disease models that compared IDH1/2^mut^ with IDH1/2^wt^ samples^48^. One dataset without publication reference with detailed information was deselected. We then selected the remaining six studies for further analysis. These studies contained at least biologically IDH1/2^mut^ triplicates and biologically IDH1/2^wt^ duplicates and the datasets of each of the studies were sufficiently significant to compile a DEG set based on an FDR-adjusted *p*-value ≤ 0.05 and an FC ≥ 1.5. In studies that employed an isogenic disease model with different IDH1/2 mutations, the raw datasets of the different IDH1/2 mutations were pooled and processed as a single IDH1/2 mutation dataset. The generated meta-analysis dataset includes GEO submissions GSE41802^22^, GSE54838^23^, GSE57002^24^, GSE88828^25^, GSE96979^21^, and GSE147223^26^. Using the same above-quoted search strategy, no additional datasets were identified in another publicly accessible repository for high-throughput functional genomics experiments^49^. The database repositories were essentially interrogated in November 2020.

### Generation of DEG sets

For four microarray GEO datasets, the binary CEL files comprising the intensity calculations were imported into Transcriptome Analysis Console (TAC) version 4.0.2.15 (Thermo Fisher Scientific, Waltham, MA). TAC includes the LIMMA (linear models for microarray data) statistical package from Bioconductor^50^. The binary CEL files were normalized in TAC and files of differentially expressed probe sets were compiled using eBayes correction in ANOVA. For the study utilizing the expression BeadChips, the normalized dataset was analyzed using the NetworkAnalyst 3.0 platform, which employs LIMMA statistics to generate differentially expressed probe sets^51^. For genes with more than one probe set in a dataset, the probe set with the highest FC was selected for further analysis; however, genes, with both significantly up- and downregulated probe sets in the same dataset, were excluded from further analysis. For the RNA-seq dataset, the publicly accessible Sequence Read Archive (SRA) datasets were downloaded from the NCBI resource^52^. We aligned the RNA-seq reads to the human reference genome assembly GRCh37 (hg19), using STAR aligner^53^. Then, the R package DESeq2 was used to normalize count data, remove outliers, determine filtering thresholds, and find genes that were significantly differentially expressed between the *IDH1*^mut^ and *IDH1*^wt^ groups^54^. Computation of the RNA-seq dataset was supported by the University High Performance Computing (Aziz Supercomputer) Center (http://hpc.kau.edu.sa). Mouse Genome Informatics (MGI), Ensembl release 101, BioMart software, and HUGO Gene Nomenclature Committee (HGNC) resources were employed to update gene IDs and/or convert mouse gene IDs to human gene IDs^55–58^. To illustrate intersecting and non-intersecting genes between the either up- or downregulated gene set of our meta-analysis and external datasets, a web-based Venn diagram tool was employed (http://bioinformatics.psb.ugent.be/webtools/Venn/).

### Ontology and pathway analysis

For further analysis of DEGs, which were based on an FDR-adjusted *p*-value ≤ 0.05 and an FC ≥ 1.5, the statistical overrepresentation test of the GO program PANTHER v. 16.0 was employed to interrogate annotation datasets in the categories of biological process, cellular component, molecular function, protein classes, and Reactome pathways^59^. The PANTHER protein class ontology comprises commonly used classes of protein functions. The Reactome pathway analysis specifies the biological relationships between interacting molecules such as nucleic acids, proteins, and compounds. For all annotation datasets, a Fisher’s exact test *p*-value < 0.05 indicated statistical significance. The BioGRID build 4.1 database was queried for protein interactors^60^. BioGRID curates protein, genetic and chemical interactions from various biomedical studies and datasets. The Ingenuity Pathway Analysis (IPA) software v. 68,752,261 (Qiagen, Hilden, Germany) was employed for further multifactorial interpretation of the gene sets. IPA utilizes the curated Ingenuity knowledge base as a reference dataset to interfere molecular relationships. Fisher’s exact test *p*-values indicated the significance of associations between analyzed dataset molecules and functional frameworks prebuilt or generated de novo by IPA. The molecule activity predictor was applied to predict expression effects/coherence of the expression effects of a molecule on other network molecules. Direct molecular relationships were used to survey the significance of fit, indicated as a score value, between molecules of uploaded gene sets and networks associated with specific functions or diseases. Direct and indirect molecular relationships were used for upstream regulator network analysis to investigate how upstream regulators affect differences in target gene expression. A z-score value indicates the activation/inhibition state of an upstream regulator.

## Supplementary Information

Supplementary Figures.

## Data Availability

The raw datasets analyzed in the study are available at the GEO repository.

## Abbreviations

α-KG: α-Ketoglutarate
AML: Acute myeloid leukemia
ANOVA: Analysis of variance
CAC: Citrate acid cycle
CEL file: Affymetrix probe results file
CpG: Cytosine-phosphate-guanine
DEG: Differentially expressed gene
EMT: Epithelial-to-mesenchymal transition
FC: Fold change
FDR: False discovery rate
GBM: Glioblastoma multiforme
G-CIMP: Glioma CpG island methylator phenotype
GEO: Gene Expression Omnibus
GO: Gene ontology
HG: Hydroxyglutarate
IDH: Isocitrate dehydrogenase
IPA: Ingenuity Pathway Analysis
LIMMA: Linear models for microarray data
lncRNA: Long non-coding RNA
mut: Mutant
RNA-seq: RNA-sequencing
SRA: Sequence Read Archive
TAC: Transcriptome Analysis Console
wt: Wild-type
