# Supplementary material for: Meta-analysis of whole-genome gene expression datasets assessing the effects of IDH1 and IDH2 mutations in isogenic disease models
Source: Sci Rep. 2022 Jan 7;12:57. doi: 10.1038/s41598-021-04214-7 (PMC8741954; doi:10.1038/s41598-021-04214-7)
Supplement: Supplementary file 1 — Supplementary Figures. [file 41598_2021_4214_MOESM1_ESM.pdf]

Meta-analysis of whole-genome gene expression datasets assessing the effects of *IDH1* and *IDH2* mutations in isogenic disease models

Hans-Juergen Schulten, Fatima Al-Adwani, Haneen A. Bin Saddeq, Heba Alkhatabi, Nofe Alganmi, Sajjad Karim, Deema Hussein, Khalid B. Al-Ghamdi, Awatif Jamal, Jaudah Al-Maghrabi, Mohammed H. Al-Qahtani

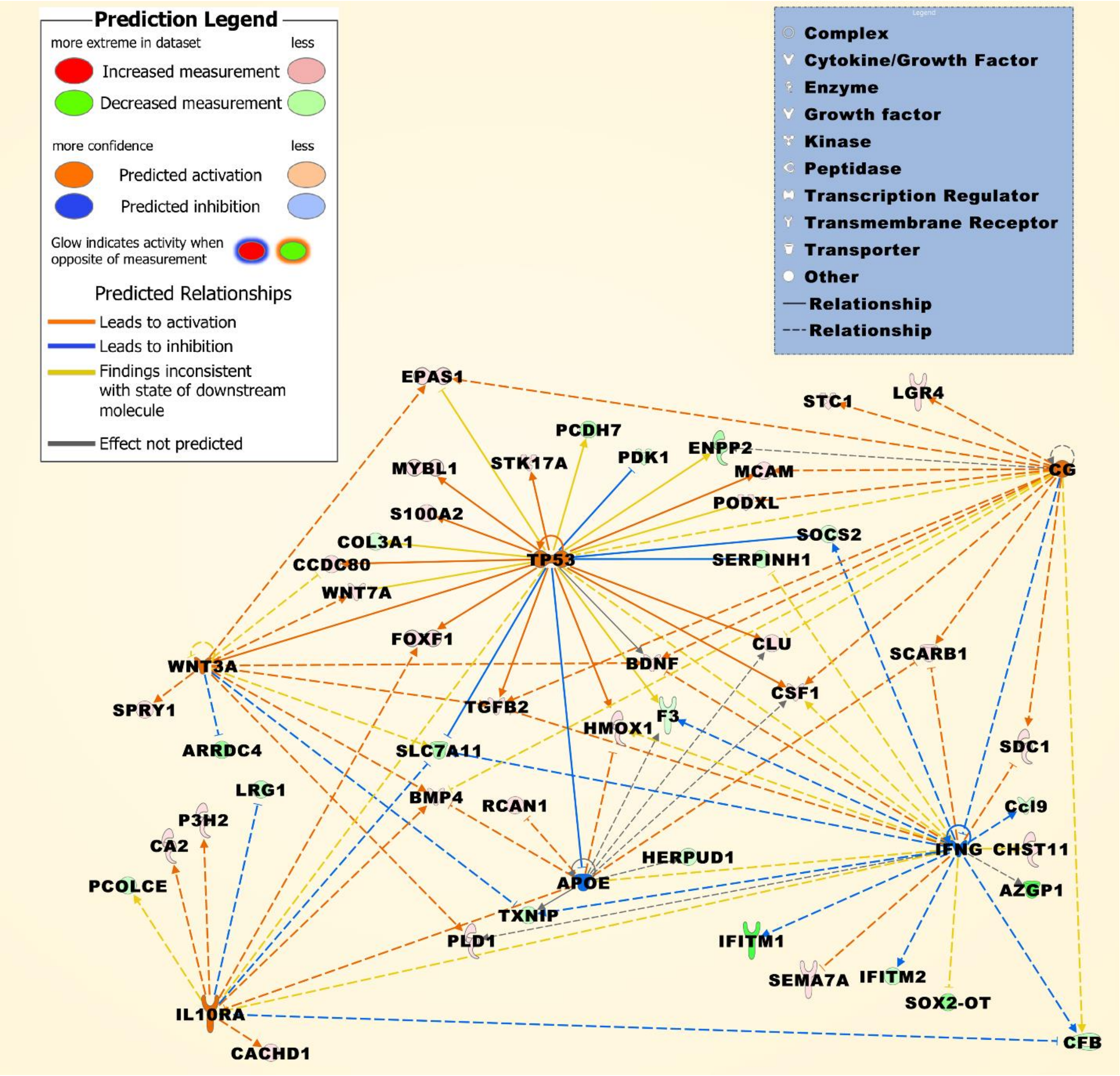

Supplementary Figure 1. A merged network based on six upstream regulators, among which CG, WNT3A, IL10RA, and TP53 were in a predicted activation state (z-score > 2) and APOE, and IFNG were in a predicted inhibition state (z-score < - 2). For all upstream regulators, the *p*-value of overlap was < 2 x 10^{-4}. Upregulated molecules from the DEG set include BDNF, BMP4, CA2, CACHD1, CCDC80, CHST11, CLU, CSF1, EPAS1, FOXF1, HMOX1, LGR4, MCAM, MYBL1, P3H2, PLD1, PODXL, RCAN1, S100A2, SCARB1, SDC1, SEMA7A, SPRY1, STC1, STK17A, TGFB2, and WNT7A. Downregulated molecules include ARRDC4, AZGP1, Ccl9, CFB, COL3A1, ENPP2, F3, HERPUD1, IFITM1, IFITM2, LRG1, PCDH7, PCOLCE, PDK1, SERPINH1, SLC7A11, SOCS2, SOX2-OT, and TXNIP. The molecule activity predictor was implemented to display further molecular effects as itemized in the prediction legend. Indirect connections are indicated with a dotted line.

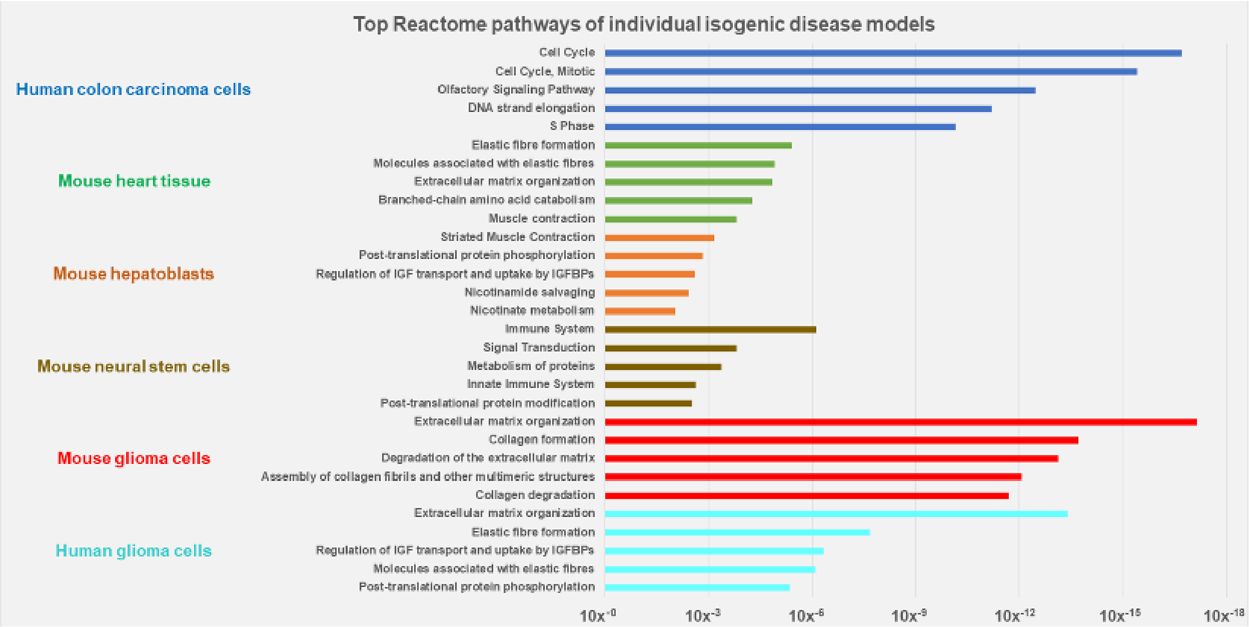

Supplementary Figure 2. Top Reactome pathways derived from the DEG set of each of the isogenic disease models. Recurrently listed pathways include extracellular matrix organization, elastic fibre formation, posttranslational protein formation, and regulation of IGF transport & uptake by IGFBPs. A Fisher's exact test *p*-value < 0.05 indicated statistical significance.

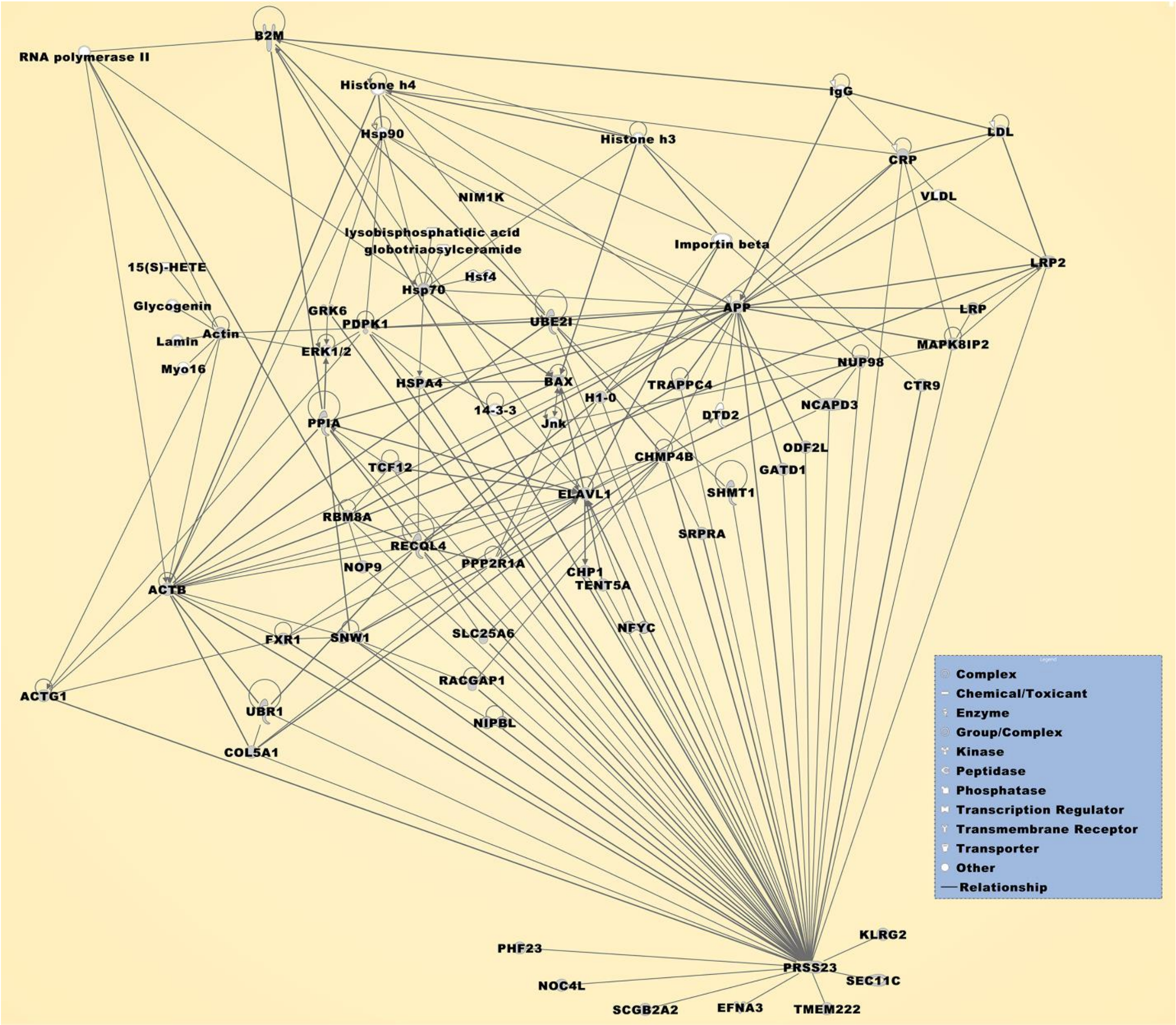

Supplementary Figure 3. The merged network, assembled with IPA, is based on the top two networks of PRSS23 protein interactors that are curated by the BioGRID database. The diseases and functions associated with the two top networks (scores 80 and 42, respectively) are related to cancer, cardiovascular disease, cellular compromise and to developmental disorder, hereditary disorder, and metabolic disease. PRSS23 protein network interactors include ACTB, ACTG1, APP, B2M, BAX, CHMP4B, CHP1, COL5A1, CRP, CTR9, EFNA3, ELAVL1, FXR1, GATD1, GRK6, H1-0, HSPA4, KLRG2, LRP2, MAPK8IP2, NCAPD3, NFYC, NIPBL, NOC4L, NOP9, NUP98, ODF2L, PDPK1, PHF23, PPIA, PPP2R1A, PRSS23, RACGAP1, RBM8A, RECQL4, SCGB2A2, SEC11C, SHMT1, SLC25A6, SNW1, SRPRA, TCF12, TENT5A, TMEM222, TRAPPC4, UBE2I, and UBR1. The most significantly overrepresented protein class associated with PRSS23 interactors comprise actin and actin-related proteins, represented by ACTB and ACTG1 (p-value < 3 x 10^{-3}). Molecular relationship factors were added from the Ingenuity knowledge base and include 14-3-3, 15(S)-HETE, Actin, DTD2, ERK1/2, globotriaosylceramide, Glycogenin, Histone h3, Histone h4, Hsf4, Hsp70, Hsp90, IgG, Importin beta, Jnk, Lamin, LDL, LRP, lysobisphosphatidic acid, Myo16, NIM1K, RNA polymerase II, and VLDL.

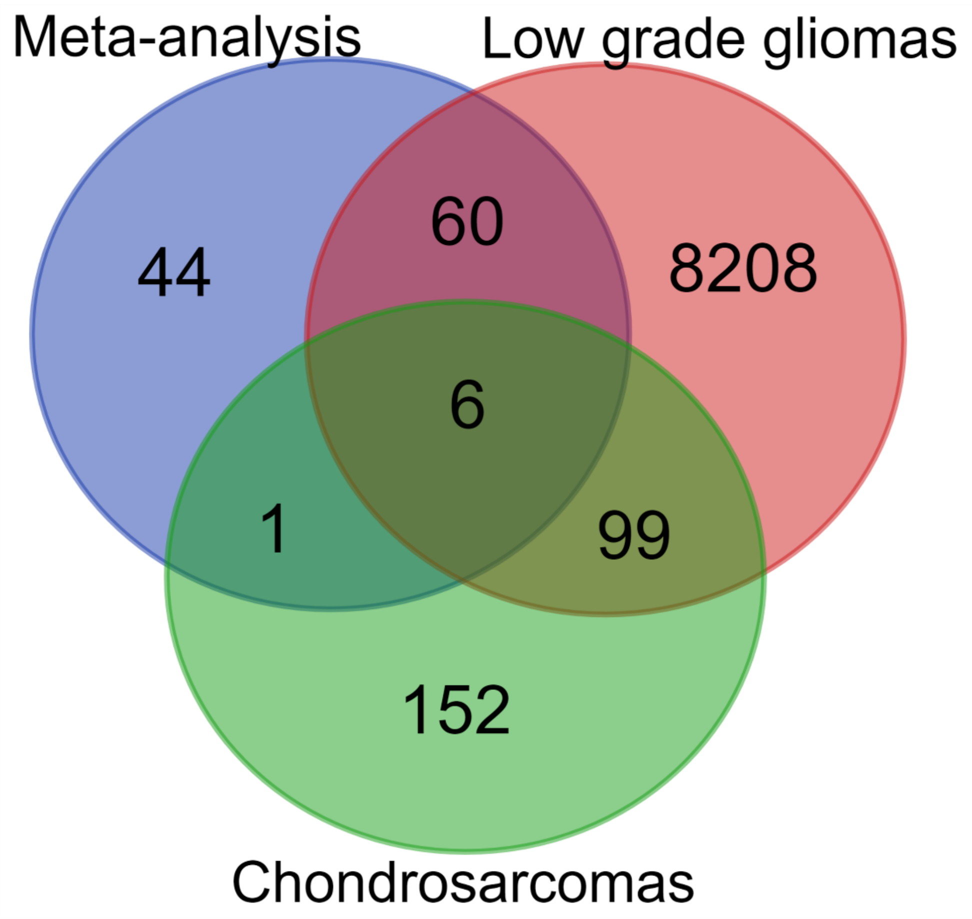

Supplementary Figure 4. A Venn diagram illustrating intersecting and non-intersecting genes that were differentially expressed between the either up- or downregulated genes of the meta-analysis and the DEGs of an Affymetrix HuGene 2.0 ST microarray analysis comprising 46 IDH^{mut} and 37 IDH^{wt} chondrosarcomas and the DEGs of a TCGA RNA-seq dataset comprising 372 IDH^{mut} and 87 IDH^{wt} low grade gliomas. The microarray dataset was downloaded from a publicly accessible repository (ArrayExpress accession number E-MTAB-7264). The normalized RNAseq gene matrix was downloaded from the Genomic Data Commons webpage (https://gdc.cancer.gov/about-data/publications/lgggbm_2016). The limma R package was used to fit a linear model on the gene matrix and to perform a pairwise comparison between the IDH^{mut} and IDH^{wt} groups. The DEGs of all three datasets were determined based on an FDR-adjusted *p*-value ≤ 0.05 and an FC ≥ 1.5. The number of DEGs is indicated in each section of the Venn diagram.
